# Tool use imagery triggers tool incorporation in the body schema

**DOI:** 10.3389/fpsyg.2014.00492

**Published:** 2014-05-30

**Authors:** Matteo Baccarini, Marie Martel, Lucilla Cardinali, Olivier Sillan, Alessandro Farnè, Alice C. Roy

**Affiliations:** ^1^Lyon Neuroscience Research Center, INSERM U1028, CNRS UMR5292, ImpAct Team, University Lyon1Lyon, France; ^2^Hospices Civils de Lyon, Mouvement et Handicap, Neuro-immersionLyon, France; ^3^Laboratory on Language, Brain and Cognition (L2C2), CNRS UMR 5304, Cognitive Sciences Institute, University Lyon 1Lyon, France

**Keywords:** tool-use, mental imagery, body representation, action, kinematics

## Abstract

Tool-use has been shown to modify the way the brain represents the metrical characteristics of the effector controlling the tool. For example, the use of tools that elongate the physical length of the arm induces kinematic changes affecting selectively the transport component of subsequent free-hand movements. Although mental simulation of an action is known to involve -to a large extent- the same processes as those at play in overt motor execution, whether tool-use imagery can yield similar effects on the body representation remains unknown. Mentally simulated actions indeed elicit autonomic physiological responses and follow motor execution rules that are comparable to those associated with the correspondent overt performance. Therefore, here we investigated the effects of the mental simulation of actions performed with a tool on the body representation by studying subsequent free-hand movements. Subjects executed reach to grasp movements with their hand before and after an imagery task performed with either a tool elongating their arm length or, as a control, with their hand alone. Two main results were found: First, in agreement with previous studies, durations of imagined movements performed with the tool and the hand were similarly affected by task difficulty. Second, kinematics of free-hand movements was affected after tool-use imagery, but not hand-use imagery, in a way similar to that previously documented after actual tool-use. These findings constitute the first evidence that tool-use imagery is sufficient to affect the representation of the user's arm.

## Introduction

Tool-use modifies our perception of the world around us. Several studies on tool-use in both healthy and brain-damaged populations have consistently reported that tool use alters our perception of space. Two main interpretations have been put forward to account for perceptual changes observed following tool-use: either space perception *per se* would be altered in such a way that far stimuli become processed as if they were nearer (Berti and Frassinetti, 2000; Maravita et al., 2001; Farnè et al., 2005; Witt and Proffitt, 2008; Holmes, 2012; Osiurak et al., 2012; Bourgeois et al., 2014), or alternatively tool-use would displace the attentional focus to the tip of the tool (Holmes et al., 2007). These effects have been related to plastic features of the multisensory processing of the peripersonal space, as identified electrophysiologically in non-human primates: in monkeys trained to retrieve distant objects with a rake, Iriki et al. (1996) revealed that visuo-tactile hand centered receptive fields appeared to extend along the tool axis (see for review, Maravita and Iriki, 2004; Cardinali et al., 2009a; Brozzoli et al., 2012). In addition, tool-use modifies the spatial metric of our own body. When asked to point to touched landmarks on their arm (middle fingertip, wrist, elbow) after using a mechanical grabber to reach and grasp objects, neurotypical participants localized these landmarks as if their touched body-parts were more distant from each other than before tool-use (Cardinali et al., 2009b, 2011a; Sposito et al., 2012; Miller et al., 2013). Most interesting for the present study, besides modifying space and body perceptual metrics, tool-use shapes our actions. The body representation we use for action (i.e., the body schema) is modified when using tools in a way such that the tool is incorporated and becomes part of our body (Baccarini and Maravita, 2013). In humans, we demonstrated that using a mechanical grabber that extends the arm's functional length by 40 cm, extends the subject's arm length representation (Cardinali et al., 2009b, 2012). Our sensorimotor system seems to be able to immediately transfer the control from the arm to the new arm + tool configuration (Van der Steen and Bongers, 2011). The motor control of free-hand reaching movements performed right after use of this tool exhibits an altered kinematics: the representation of an elongated arm in the body schema translates in the later occurrence and reduced amplitude of some kinematics events (acceleration, velocity, deceleration peaks). Such changes in motor control of the arm have been considered as the key kinematics signatures for the incorporation of the tool into the body schema (Cardinali et al., 2011b) and revealed the latter is a highly plastic representation that quickly builds-up on previous experience.

Strikingly, mere mentally simulated motor experiences are sufficient in some cases to trigger subsequent actions modifications and athletes commonly use motor imagery to improve their performance (Driskell et al., 1994; Roure et al., 1999). Motor imagery might be sufficient to acquire functional object knowledge, however the built representations have been shown to be less detailed than when experiencing actual movement with the object (Macuga et al., 2012; Paulus et al., 2012). Moreover, brain areas recruited to perform actual or imagined movement execution are not strictly overlapping (Imazu et al., 2007; see for review Dietrich, 2008). Nevertheless a contagion from movement imagery to movement execution is possible as both evolve on a similar time-scale and follow very similar biomechanical rules. Motor imagery follows so faithfully the constraints imposed to the motor system that the postural adjustments normally accompanying a voluntary reaching movement while standing up are also present in an imagined reaching situation (Boulton and Mitra, 2013). Execution time of mentally simulated movements has been shown to be comparable in duration to actually executed movements (Papaxanthis et al., 2002; for review, see Jeannerod and Frak, 1999; Guillot and Collet, 2005). An important constraint of the motor system is the speed accuracy trade-off known as Fitts law (1954). According to this law, increasing the velocity of execution of an action leads to decrease in accuracy, and conversely, increasing the accuracy demands increases the time needed to perform the task. Several studies have demonstrated that Fitt's law holds in motor imagery, imagined movement times linearly increasing with task difficulty (Decety and Jeannerod, 1995; Maruff et al., 1999). In a prehension task paradigm, Frak et al. (2001) had subjects to physically or mentally grasp a cylinder between the index and thumb while varying the orientation of the axis formed by the opposed fingertips on the object, the so-called opposition axis. When free to adopt a natural finger positioning on the object, subjects typically tend to keep the opposition axis invariant from trial to trial, as changing it determines an additional cost on the musculo-articulatory system (Paulignan et al., 1997). Frak et al. (2001) elegantly demonstrated that prehension movements requiring different pre-determined orientations of the opposition axis induce similar modulations of movement time for both physically executed and imagined movements. Recently, Jacobs et al. (2010) used a similar paradigm to investigate free-hand grasping and grasping with a handheld tool. Subjects' performance during mental imagery respected the bio-mechanical constraints imposed by the tool during real movement execution (see also Rieger and Massen, 2014). Tool-use imagery has been less explored but is known to modify space perception as tool execution does (Witt and Proffitt, 2008; Davoli et al., 2012; Gabbard and Caçola, 2013) and to follow Fitt's law (Macuga et al., 2012). Most recently it has been reported that expert tool-users are sensitive to the held tool during imagery whereas naive tool-users are not (Bisio et al., 2014).

On the one hand, thus, evidence from real tool-use indicates that it modifies the kinematics of subsequent free-hand movements as if they were performed with a longer arm; on the other hand, mental imagery of tool-use seems to reproduce tool-use execution quite accurately. Taken together, these findings raise the question of whether mere tool-use imagery is sufficient to modify the representation of the arm's length. To answer this question we designed an experiment in which the rationale was the following: if imagining using the same mechanical grabber that extends the arm's length by 40 cm (Cardinali et al., 2009a,b, 2012) is sufficient for this tool to be incorporated into the body schema and thus increases the subject's represented arm length, then the real execution of free hand prehension movements subsequent to tool-use imagery should display those kinematics signatures that we observed after actual tool-use. Since motor imagery is known to be modulated by task difficulty, varying task difficulty is an efficient way to control that motor imagery was properly performed (Lotze and Halsband, 2006). We therefore applied the paradigm introduced by Frak et al. (2001), and manipulated the orientation of the opposition axis to be used to grasp a cylinder in order to vary movement's difficulty. In different sessions separated by one day, participants were required to perform prehension movements toward objects with different oppositions axes before and after having mentally simulated these movements with their free hand (as a control), or using the mechanical grabber.

## Materials and methods

### Participants

Sixteen neurologically healthy subjects (8 male; mean age 22.4 years; *SD*: 3.7; range from 18 to 32) participated in the study. All were right-handed and had normal or corrected-to-normal vision. All participants gave written informed consent to participate in the study, which was approved by the local ethics committee and conformed to the Helsinki Declaration.

### Apparatus and procedures

**Figure 1:**
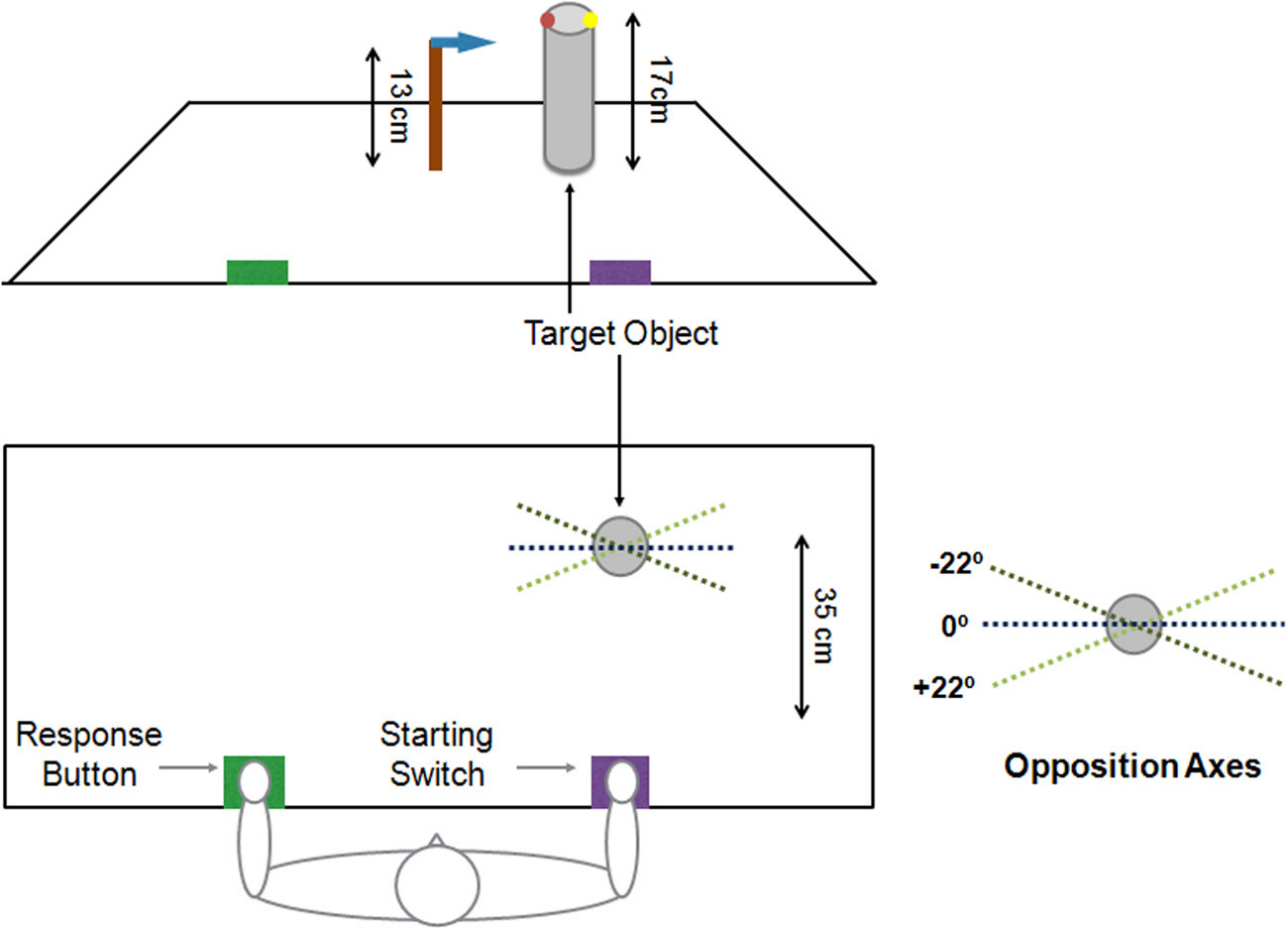
**Schematic representation of the experimental set up from the subject's point of view (upper panel) and from above (lower panel)**. Subjects placed their right hand on a starting switch (purple) and the left hand on a response button (green). The target object was a cylinder, located 35 cm from the starting point. On its upper side were two colored dots indicating the location of the fingers (red for the thumb and yellow for the index); the line between these two dots constituted the opposition axis, which could be of three orientations: −22°, 0° and +22°. On the left an arrow indicated the height to which the object should be lifted.

Participants were comfortably seated in front of a table with the right hand closed in a pinch-shaped grip on a switch. The left hand, palm down, was pressing a response button. The target object was a plastic cylinder (5 cm in diameter and 17 cm height) placed on the table at a distance of 35 cm along the sagittal axis, in line with subjects' right shoulder. Two colored dots on the upper edge of the cylinder marked the grasp landing positions required for the tips of the thumb (red) and index fingers (yellow). The virtual line connecting these two points of contact determined the Opposition Axis (OA) of the grip. At the beginning of each trial, the cylinder was presented with one of three possible OA, namely −22°, 0° and +22° with respect to the subject trunk. Each OA was presented an equal number of times in a pseudo-randomized order. A horizontal arrow was taped at 13 cm of height from the table on a wooden block, located about 10 cm to the left of the cylinder and served to indicate the height at which the participants had to lift the object (Figure 1).

**Figure 2:**
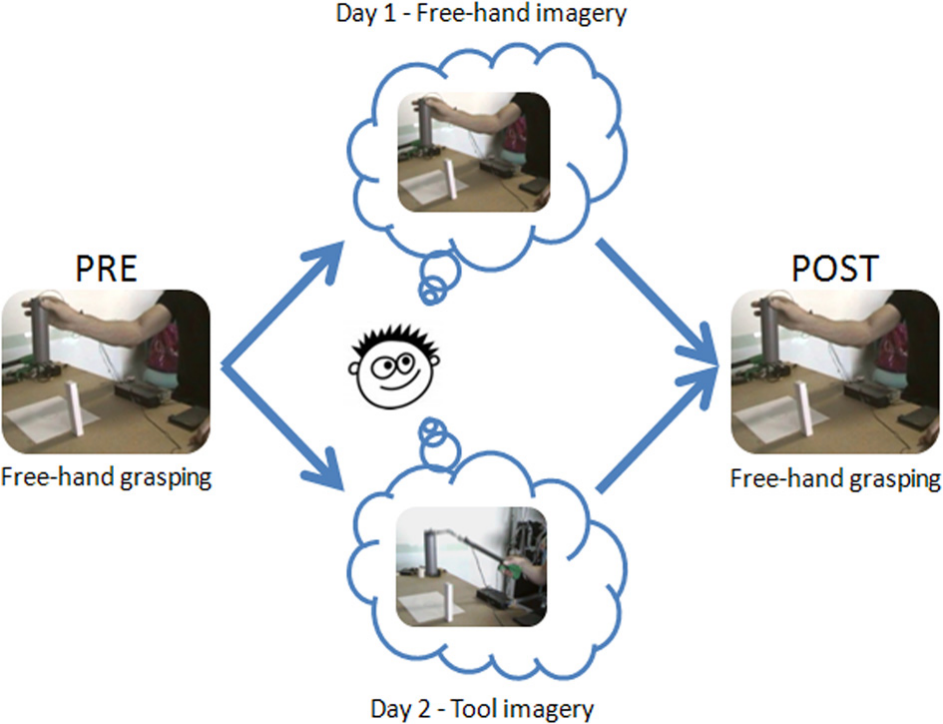
**Experimental design and procedure**. The experiment took place during two consecutive days, each including three experimental sessions: Pre-imagery free-hand execution session (18 trials), Motor Imagery session (54 trials), Post-imagery free-hand execution session (18 trials).

The experiment consisted of three tasks, each presented over two consecutive days: Pre-imagery free-hand grasping task (18 trials), Motor Imagery task (54 trials), and Post-imagery free-hand grasping task (18 trials). During the Pre- and Post-imagery free-hand grasping task, participants were required to reach, grasp and lift the target object up to the arrow with their right hand (see Figure 2). They were instructed to grasp the object using a precision grip by placing their thumb and index fingertips on the respective colored dots. Once the trial was performed, participants got back to the starting position, closed their eyes and waited for an acoustical “go” signal to open their eyes and perform the next trial.

During the Motor imagery task of day 1, subjects were required to imagine using their right hand to reach for, grasp and lift the cylinder up to the height indicated by the arrow. They were instructed to wait for an acoustical go signal to open their eyes and start imagining performing the task. Participants had to raise their left hand to release the switch as they started their imagery trial, and to put their hand back down to press the switch once it was accomplished (i.e., the object was lifted at the height indicated by the arrow). Participants' right hand was kept still on the right switch during the whole duration of the imagery task. Two pauses were planned after 18 trials and 36 trials respectively. Other pauses were delivered if required. During pauses participants were allowed to open and close the fingers of their right hand and to move their arms. The Motor imagery task of day 2 was identical to that of day 1, except that subjects had to imagine performing the prehension movement with a grabber they were holding still in their right hand. The grabber was constituted of an ergonomic handle (9 cm) fitted with a lever, a 33-cm-long rigid shaft, and a “hand” with two articulated fingers (10 cm). Squeezing the lever (vertically) made the “fingers” of the tool close (horizontally). The grabber used here was identical to that used in previous work documenting effects of actual too-use on subsequent free-hand kinematics (Cardinali et al., 2009b). During the whole duration of the imagery task the “tool fingers” were kept in a pinch grip posture on the start switch. During pauses, subjects were allowed to move the arm, but could not drop the tool. In order to be able to imagine using the grabber, at the end of day 1 subjects were familiarized with the tool by performing 18 grasping trials (6 for each opposition axis). Tool-use imagery never took place on day 1 to avoid potentials tool integration effects to carry over on day 2.

### Kinematic recording

Three infrared light emitting diodes (IREDs) were placed on the subjects' right hand: on the medial lower corner of the thumb nail, on the lateral lower corner of the index finger nail and on the skin proximal to the styloid process of the radius at the wrist. The reaching component of the movement was characterized by the wrist marker displacement, while the grip component was characterized by the thumb and index displacement. Spatial localization of the markers was recorded with an Optotrak 3020 (Northern Digital Inc; sampling rate: 200 Hz; 3D resolution: 0.01 mm at 2.25 m distance). Analyzed parameters included latencies and amplitudes of acceleration, velocity and deceleration peaks for the transport component, and latency and amplitude of the maximum grip aperture for the grip component. The total movement duration of imagined movements (from release to press of the left response button, corresponding to the same events of actual movements) was also extracted.

### Statistical analysis

To assess the effect of the OA on imagined movements, subjects' average imagined movement durations (MD) were submitted to a repeated measure ANOVA with Effector (hand/tool) and Opposition Axis (−22°/0°/+22°) as within-subject factors. In order to establish the effect of motor imagery with the tool on subsequent free-hand movements, we performed a repeated measure ANOVA on movement kinematic parameters with type of Imagery (hand/tool), Session (pre / post imagery) and Opposition Axis (−22°/0°/+22°) as within-subject factors. When necessary, Newman-Keuls *post-hoc* test were used.

## Results

### Movement durations during motor imagery

**Figure 3:**
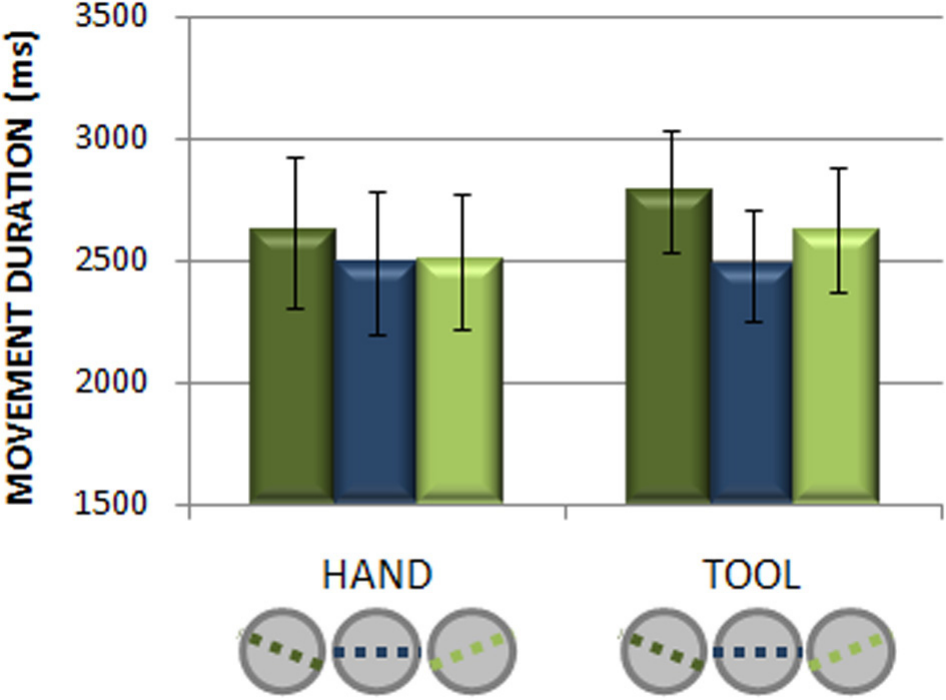
**Imagined movement duration**. The graph displays the average imagined movement duration as a function of effector (hand and tool) and orientation of the Opposition Axis (−22°; 0°; +22°). Bar graphs illustrate mean values for each parameter ±1 s.e.m.

As shown in Figure 3, the analysis revealed no significant difference between hand and tool imagined movement durations [*F*_(1, 15)_ = 0.60, *p* = 0.45; *MD* = 2538 vs. 2633 ms]. A main effect of Opposition Axis [*F*_(2, 30)_ = 16.0, *p* < 0.001; η^2^_*p*_ = 0.52] highlighted that the most difficult OA(−22°) required longer performance time (*MD* = 2702 ms) compared to the other orientations (0° *MD* = 2489 ms; +22° *MD* = 2565 ms, all *p*-values < 0.002), which tended to differ between them (*p* = 0.055). The interaction between Effector and Opposition Axis almost reached significance [*F*_(2, 30)_ = 3.0, *p* = 0.065], potentially suggesting that OA may have a slightly different impact on tool and free-hand imagery.

Taken together these results highlight the difficulty raised by the most unnatural opposition axis (−22°) irrespective of the used effector, indicating that participants performed free-hand and tool imagery tasks reliably.

### Effect of free-hand vs. tool motor imagery on subsequent free-hand movements

**Figure 4:**
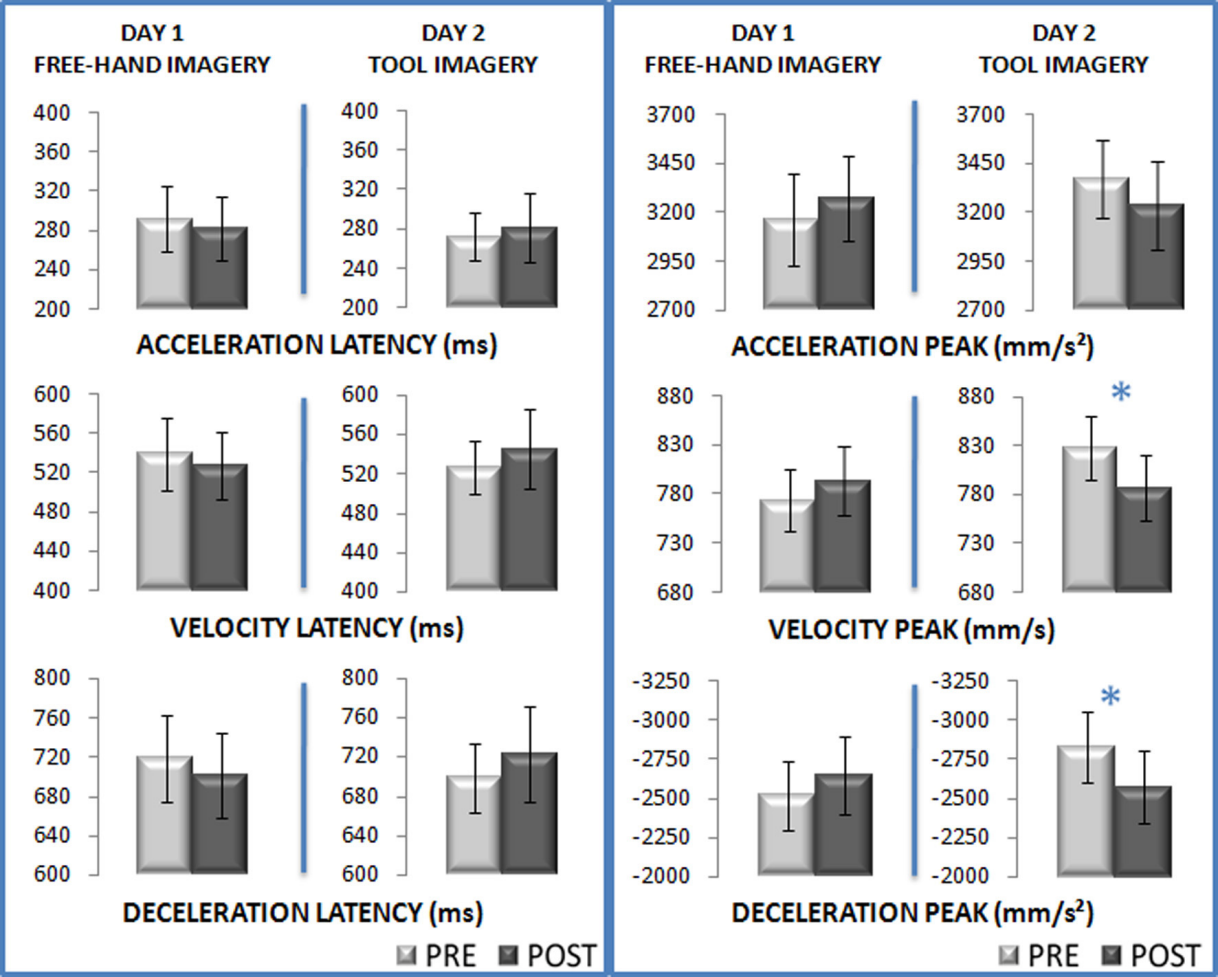
**Tool-use imagery modifies free-hand movement kinematics**. Bar graphs illustrate mean values for each parameter ±1 s.e.m. Asterisks denote significant differences from Newman-Keuls *post-hoc*.

To investigate the effects of tool-use imagery on the subsequent free-hand movement execution, the following section focuses on the critical interaction between the factors type of Imagery (hand vs. tool) and Session (pre vs. post imagery) across the kinematic parameters of the transport and grasping components (see Tables 1, 2 for an exhaustive report of the statistical results and means respectively). Two of such interactions were found to be significant, for the wrist velocity peak [*F*_(1, 15)_ = 11, *p* < 0.01; η^2^_*p*_ = 0.42] and the deceleration peak [*F*_(1, 15)_ = 9.76, *p* < 0.01; η^2^_*p*_ = 0.39; see Figure 4]. Free-hand imagery did not induce any significant modifications on the subsequent movements' kinematics (velocity peak: pre: 773 mm/s vs. post: 793 mm/s, *p* = 0.30; deceleration peak: pre: −2511 mm/s^2^ vs. post: −2632 mm/s^2^, *p* = 0.28). The pre imagery session of day 1 differed from that of day 2, in that subjects reached higher velocity and deceleration peaks -before motor imagery- in day 2 as compared to day 1 (all *p* < 0.01), compatible with some practice effects. Critically, participants' free-hand movements performed after tool-use imagery exhibited significantly decreased wrist velocity peak (pre 827 mm/s vs. post: 785 mm/s, *p* < 0.02) and deceleration peak (pre: −2829 mm/s^2^ vs. post: −2569 mm/s^2^, *p* < 0.04) with respect to those performed before tool imagery. As expected, no significant interaction was found on the kinematic parameters of the grasping component.

**Table 1:** **Main effects and interactions observed for the ANOVA performed on each kinematic parameter**.

	**Imagery Type (hand/tool)**				**Session (pre/post)**				**Opposition axis (OA)**				Type^*^Session			
**Parameters**	***df***	***F***	***P***	**η^2^**	***df***	***F***	***P***	**η^2^**	***df***	***F***	***P***	**η^2^**	***df***	***F***	***P***	**η^2^**
Acceleration Latency	(1, 15)	0.945	0.346	0.059	(1, 15)	0.075	0.788	0.005	(2, 30)	1.74	0.193	0.104	(1, 15)	0.409	0.532	0.027
Acceleration Peak	(1, 15)	0.962	0.342	0.060	(1, 15)	0.084	0.775	0.006	**(2, 30)**	**6.63**	**0.004**	**0.306**	(1, 15)	1.44	0.250	0.087
Velocity Latency	(1, 15)	0.082	0.779	0.005	(1, 15)	0.007	0.933	0.001	(2, 30)	0.419	0.661	0.027	(1, 15)	1.00	0.332	0.063
Velocity Peak	(1, 15)	2.54	0.132	0.145	(1, 15)	0.70	0.417	0.044	**(2, 30)**	**5.50**	**0.009**	**0.268**	**(1, 15)**	**11.0**	**0.005**	**0.423**
Deceleration Latency	(1, 15)	0.013	0.911	0.001	(1, 15)	0.051	0.824	0.004	(2, 30)	1.43	0.257	0.093	(1, 15)	2.07	0.172	0.129
Deceleration Peak	(1, 15)	2.09	0.169	0.122	(1, 15)	0.314	0.584	0.020	(2, 30)	1.26	0.298	0.078	**(1, 15)**	**9.76**	**0.007**	**0.394**
MGA Latency	(1, 15)	3.56	0.080	0.203	(1, 15)	0.013	0.910	0.001	(2, 30)	1.80	0.184	0.114	(1, 15)	0.623	0.443	0.043
Maximum Grip Aperture	(1, 15)	0.469	0.506	0.003	(1, 15)	2.24	0.159	0.147	(2, 30)	0.144	0.867	0.001	(1, 15)	0.276	0.608	0.002
	Type^*^OA				Session^*^OA				Type^*^Session^*^OA							
**Parameters**	***df***	***F***	***P***	**η^2^**	***df***	***F***	***P***	**η^2^**	***df***	***F***	***P***	**η^2^**				
Acceleration Latency	**(2, 30)**	**5.11**	**0.012**	**0.254**	(2, 30)	2.33	0.115	0.134	(2, 30)	0.138	0.871	0.009				
Acceleration Peak	(2, 30)	0.627	0.541	0.040	(2, 30)	0.654	0.527	0.042	(2, 30)	0.098	0.907	0.006				
Velocity Latency	(2, 30)	0.695	0.507	0.044	**(2, 30)**	**5.77**	**0.008**	0.**278**	(2, 30)	0.031	0.969	0.002				
Velocity Peak	(2, 30)	0.29	0.751	0.019	(2, 30)	1.03	0.369	0.064	(2, 30)	0.03	0.972	0.002				
Deceleration Latency	(2, 30)	0.504	0.609	0.035	(2, 30)	2.52	0.098	0.153	(2, 30)	0.703	0.504	0.048				
Deceleration Peak	(2, 30)	1.55	0.229	0.094	(2, 30)	0.125	0.883	0.008	(2, 30)	0.618	0.546	0.040				
MGA Latency	(2, 30)	1.11	0.344	0.073	(2, 30)	1.61	0.217	0.103	(2, 30)	0.207	0.814	0.015				
Maximum Grip Aperture	(2, 30)	1.68	0.207	0.114	**(2, 30)**	**6.42**	**0.005**	**0.330**	(2, 30)	1.62	0.217	0.111				

*MGA, maximum grip aperture. Significant p values (<0.05) are reported in bold*.

**Table 2:** **Main values ± 1 s.e.m. of each kinematic parameter according to the full factorial design**.

	**Hand**						**Tool**					
	**Pre**			**Post**			**Pre**			**Post**		
	**OA**			**OA**			**OA**			**OA**		
	**−22°**	**0°**	**22°**	**−22°**	**0°**	**22°**	**−22°**	**0°**	**22°**	**−22°**	**0°**	**22°**
Acceleration latency (ms)	297 ± 26	291 ± 27	290 ± 26	280 ± 32	283 ± 27	277 ± 23	259 ± 20	273 ± 18	288 ± 17	263 ± 30	288 ± 30	297 ± 27
Acceleration peak (mm/s^2^)	3147 ± 205	3250 ± 177	3077 ± 187	3305 ± 198	3303 ± 184	3203 ± 178	3440 ± 175	3410 ± 167	3254 ± 147	3294 ± 199	3269 ± 196	3104 ± 179
Velocity latency (ms)	553 ± 34	531 ± 31	538 ± 29	527 ± 33	533 ± 30	521 ± 27	524 ± 26	528 ± 24	529 ± 20	551 ± 39	545 ± 39	545 ± 33
Velocity peak (mm/s)	775 ± 28	782 ± 28	761 ± 25	804 ± 29	797 ± 34	777 ± 33	833 ± 30	831 ± 29	817 ± 30	790 ± 29	791 ± 31	775 ± 34
Deceleration latency (ms)	741 ± 36	697 ± 34	721 ± 36	686 ± 37	702 ± 36	714 ± 40	701 ± 33	689 ± 32	707 ± 27	726 ± 43	717 ± 45	735 ± 46
Deceleration peak (mm/s^2^)	−2452 ± 174	−2625 ± 197	−2455 ± 184	−2531 ± 218	−2703 ± 221	−2663 ± 222	−2778 ± 194	−2860 ± 181	−2850 ± 209	−2543 ± 184	−2591 ± 212	−2574 ± 231
MGA latency (ms)	883 ± 49	837 ± 49	849 ± 45	854 ± 55	838 ± 47	832 ± 51	828 ± 42	815 ± 43	815 ± 36	837 ± 62	836 ± 67	841 ± 61
Maximum grip aperture (mm)	102 ± 2	100 ± 2	99 ± 2	97 ± 2	99 ± 2	98 ± 2	101 ± 3	99 ± 3	100 ± 3	95 ± 3	96 ± 3	98 ± 3

*MGA, maximum grip aperture*.

## Discussion

Here we investigated the effects of tool-use imagery vs. free hand imagery on subsequent free-hand grasping movements. As movement imagery is sensitive to task difficulty we asked participants to conform their final grip to three opposition axes of varying difficulty. In line with our expectations, the opposition axes differently taxed imagined movement durations, thus confirming that participants successfully engaged in the imagery tasks. Our analysis then focused on the differences of free-hand imagery vs. tool-use imagery on subsequent movements. While free hand grasping imagery did not affect actual free-hand movements, the latter movements performed after tool-use imagery were characterized by significant decrease of both wrist velocity and deceleration peaks. Together with previous findings from our group, these results indicate that imagery of tool-use may be sufficient to update the representation of the arm length used to execute free-hand movements. We have indeed reported previously that using a tool to grasp an object modifies the kinematics of subsequent free-hand movements as if the participant performed object prehension with a longer arm (Cardinali et al., 2009b), and we proposed that these kinematic modifications are the fingerprint of the tool incorporation in the body schema (Cardinali et al., 2011a,b, 2012). Here we investigated whether tool-use imagery could be sufficient to induce such modifications of the body schema. While imagery has been largely explored in psychology and cognitive sciences, tool-use imagery has become a field of investigation only recently. Rieger and Massen (2014) have examined how different tools translate in different tool imagery performances by requiring participants to color a rectangle using pens with different thicknesses. As it was the case for physically executed actions, imagined actions were influenced by the pen's thickness, the thinnest one giving rise to longer movement times to fill-up the rectangle. In the same vein, Macuga et al. (2012) reported that despite some inaccuracy, the Fitt's law holds for movements imaginarily performed with tools.

To make a step forward, here we tested whether tool-use imagery effects, besides influencing ongoing performance *during* the tool-use imagery task, can last sufficiently to modify actual movements performed *afterwards*, without the tool. We first aimed to ensure that imagery was accurately performed. To this aim, we varied the difficulty of an object prehension task by requiring participants to grasp a cylinder putting their index and thumb or the tool's “fingers” in predetermined positions on the cylinder, thus creating different orientations of the opposition axis(OA) between the fingertips. Our findings confirm and extend those of Frak et al. (2001) as we show that the −22° orientation of the OA is the most difficult and hence time consuming one, irrespective of whether movements were imagined with the hand or the tool.

When considering the effects of tool imagery on subsequent movements, our results make a considerable step further by demonstrating that tool-use imagery is sufficient to warrant tool incorporation in the body schema (i.e., the representation the brain uses to plan and execute actions). When comparing free hand movements performed before and after tool-use imagery, movement kinematics presented wrist velocity and deceleration peaks of decreased amplitude. Previously, after physical tool-use, we reported such reductions in amplitude for the very same kinematic parameters, accompanied by protracted latencies and discussed these kinematics modifications as the hallmark of tool incorporation in the body schema (Cardinali et al., 2009b, 2012). Similar to previous work, here the direction of the changes triggered by tool-use imagery on the subsequent movement kinematics (i.e., the reduction of maximum velocity and deceleration peaks) is compatible with a change of the represented length of the arm in the direction of its elongation. Compared to short-arm people, long(er)-armed participants naturally tend to perform the same grasping action with reduced velocity and deceleration peaks. For such movements, they also tend to display longer latencies of these parameters (see supplemental data in Cardinali et al., 2009b). In the present study, a relatively brief tool-use imagery task appeared sufficient to reduce the maximal amplitude of transport component parameters, thus suggesting profound consequences for real movements, from imagined movement execution. In contrast to our previous work, the latencies of the same parameters were not significantly modified by tool-use imagining suggesting that although very similar, tool-use imagery is not in all respects identical to actual tool-use execution. Nevertheless the modifications in motor control did replicate those found after actual tool-use both in the direction (i.e., reduction) and specificity, affecting selectively the transport component parameters and leaving the grasping ones unaltered (Cardinali et al., 2009b, 2012).

Noteworthy, the modifications on real hand movements induced by tool-use imagery unambiguously points to a tool incorporation in the body schema (e.g., reduced wrist velocity) and as such differ from the learning effects typically reported after mental practice (increased performance due to increased velocity). This observation finds additional support in the results of free-hand imagery performed in day 1. Indeed, normal subjects are by essence experts in performing manual prehension and hence mental training with the very same effector was ineffective in triggering any significant kinematic modification of subsequent executed movements (Allami et al., 2007). Moreover, the pre imagery session of day 2 as compared to that of day 1 displayed increased velocity and deceleration peaks, an effect that is exactly opposite to the one observed after tool-use imagery.

Finally, potential limitations of our study need to be addressed. First, the lack of execution session with the tool, before motor imagery, prevented us from directly comparing execution and imagery movement duration with the tool. Our main aim was not to compare tool execution and tool imagery (see Rieger and Massen, 2014 and Macuga et al., 2012 for this comparison), rather our study focused on tool-use vs. free-hand imagery effects on subsequent free-hand movements. A second potential limitation arises from the fact that to avoid potential carry-over effects free-hand imagery and tool-use imagery were not counterbalanced, tool-use imagery occurring always on day 2 after hand imagery. The post-test performed on day 2 is thus the fourth time subjects executed the free-hand grasping task. One could have expected a facilitation effect similar to that observed between the pre session of day 1 and 2; by contrast, velocity and deceleration peaks decreased after tool-use imagery, an effect that is thus compatible with our previous results obtained after physical tool-use. Third, it might have been of interest to directly compare the consequences of both tool-use execution and tool-use imagery. Since we used the very same paradigm and grabber as the one we used for evaluating the effects of tool use execution (Cardinali et al., 2009b, 2012), the results obtained here nevertheless point to some differential effect of imagined vs. real tool-use, as the velocity and deceleration peaks, but not the latencies of these parameters, were affected by tool-use imagery.

To conclude, tool-use imagery not only adheres to most of the physical rules of actual movement execution, but has protracted consequences on the real execution of movements performed afterwards without the tool that are readily understandable as the product of previous tool incorporation.

### Conflict of interest statement

The authors declare that the research was conducted in the absence of any commercial or financial relationships that could be construed as a potential conflict of interest.
